# Baseline Audit of Smoke-Free Policy Compliance on Older Adult Psychiatric Inpatient Wards

**DOI:** 10.1192/bjo.2026.11730

**Published:** 2026-06-30

**Authors:** Lucy Revitt, Akpevwe Egbegbedia

**Affiliations:** 1 Blackpool Teaching Hospital NHS Foundation Trust, Blackpool, United Kingdom; 2 Lancashire and South Cumbria Foundation Trust, Blackpool, United Kingdom

## Abstract

**Aims:**

To assess compliance with Trust Smoke-Free standards on older adult psychiatric wards, specifically: documentation of smoking status on admission; timely initiation of nicotine replacement therapy (NRT); regular multidisciplinary team (MDT) review of tobacco dependence treatment; and referral to the Trust Tobacco Dependency Team. The audit also aimed to identify potential age - or diagnosis-related inequities in assessment and treatment.

**Methods:**

A cross-sectional baseline clinical audit was conducted across four older adult psychiatric inpatient wards. All inpatients present on 21/11/2025 were included (n=53). Data were extracted from electronic patient records (RiO), the electronic prescribing system (EPMA), and MDT documentation. Compliance was measured against four Trust Smoke-Free standards. Smoking status documentation was assessed across all admissions, while treatment, review, and referral standards were assessed in patients with documented tobacco dependence (n=6).

**Results:**

Smoking status was documented during admission clerking in 31% of patients (16/53). Among identified smokers, compliance with prescribing tobacco dependence treatment within 30 minutes of admission was 0%. Although 83% of smokers were eventually prescribed NRT, all prescriptions occurred more than five hours after admission, and 17% of smokers were never prescribed NRT. There was 0% compliance with regular MDT review of tobacco dependence treatment. Referral to the Tobacco Dependency Team within 24 hours occurred in only 17% of smokers.

**Conclusion:**

This audit demonstrates poor baseline compliance with Smoke-Free standards on older adult psychiatric wards, particularly in the identification and timely management of tobacco dependence. The findings highlight risks of diagnostic overshadowing and avoidable behavioural disturbance in older adult psychiatry. Targeted quality improvement interventions focusing on admission clerking, MDT review, and referral pathways are required to promote equitable, person-centred care and an equal standard of care. A re-audit is planned following implementation of these interventions.

